# Expression of Galectin-9-related immune checkpoint receptors in B-cell acute lymphoblastic leukemia

**DOI:** 10.22038/IJBMS.2023.73159.15901

**Published:** 2023

**Authors:** Armin Akbar, Hossein Asgarian-Omran, Reza Valadan, Mohammad-Mehdi Dindarloo, Ahmad Najafi, Amir Kahrizi, Arash Poursheikhani, Hossein Karami, Mohammad Naderi, Shayan Sabeti, Mohsen Tehrani

**Affiliations:** 1 Department of Immunology, School of Medicine, Mazandaran University of Medical Sciences, Sari, Iran; 2 Molecular and Cell-Biology Research Center, Mazandaran University of Medical Sciences, Sari, Iran; 3 Department of Biostatistics and Epidemiology, Mazandaran University of Medical Sciences, Sari, Iran; 4 Legal Medicine Research Center, Legal Medicine Organization, Tehran, Iran; 5 Department of Medical Genetics, Faculty of Medicine, Mashhad University of Medical Sciences, Mashhad, Iran; 6 Department of Hematology and Oncology, Imam Khomeini Hospital, Mazandaran University of Medical Sciences, Sari, Iran; 7 Thalassemia Research Center (TRC), Hemoglobinopathy Institute, Mazandaran University of Medical Sciences, Sari, Iran

**Keywords:** Acute lymphoblastic – leukemia, Gelectin-9, HAVCR2, Immune checkpoint – receptor, T-cell exhaustion, TIM-3, VISTA

## Abstract

**Objective(s):**

Exhausted CD8+ T-cells over-express immune checkpoint receptors (ICRs), which interact with their ligands on malignant cells. However, some ICRs have been reported to be expressed on both T-cells and tumor cells, including V-domain immunoglobulin suppressor of T cell activation (VISTA), Galectin-9, and T-cell immunoglobulin mucin-3 (TIM-3). We aimed to evaluate the mRNA expression of VISTA, Galectin-9, and TIM-3 on CD8+ T-cells and leukemic cells in B-cell acute lymphoblastic leukemia (B-ALL).

**Materials and Methods:**

Samples were obtained from 26 untreated B-ALL patients and 25 control subjects. CD8+ T-cells were isolated using Magnetic Activated Cell Sorting (MACS). Relative gene expression was then evaluated by qRT-PCR with specific primers for VISTA, Galectin-9, and TIM-3. Also, the mRNA expression profile and clinical data of 154 B-ALL patients were obtained from the TARGET.

**Results:**

mRNA expression of Galectin-9 on CD8+ T-cells in B-ALL patients was significantly lower than those in the control group (*P*=0.043), while VISTA expression was not significantly different between the two study groups (*P*=0.259). Besides, TIM-3 expression was significantly higher in B-ALL patients than in the control group (*P*<0.001). Also, data obtained from TARGET showed that the relapse incidence was not significantly different between patients with high and low expression of Galectin-9 and TIM-3 in leukemic cells (*P*=0.360 and *P*=0.655, respectively).

**Conclusion:**

Collectively, gene expression results suggest an important role for TIM-3, but not VISTA and Galectin-9, in B-ALL and it seems that TIM-3 could be a candidate for immune checkpoint therapy.

## Introduction

B-cell acute lymphoblastic leukemia (B-ALL) is a lethal malignancy characterized by clonal proliferation of B-cell precursors that typically affects children younger than six years of age, while it can also be found in older children and adults (1). Besides chemotherapy, radiation therapy, and hematopoietic stem cell transplantation (HSCT) (2), many studies have focused on immunotherapeutic strategies. In chronic infections and cancers, T-cells become exhausted (3), where they over-express immune-checkpoint receptors (ICRs), such as Cytotoxic T-Lymphocyte–Associated Antigen 4 (CTLA-4), Programmed Death-1 (PD-1), Lymphocyte Activation Gene-3 (LAG-3), and T-cell immunoreceptor with immunoglobulin and ITIM domain (TIGIT), which bind their ligands and lead to defects in proliferation, cytokine production, and cytotoxicity of T-cells. Immune checkpoint blockade (ICB) therapy using CTLA-4 and PD-1 is exhibiting noticeable efficacy in several solid tumors; nevertheless, some tumors remain largely resistant to these treatments (3-5), thus requiring further investigation into targeting new immune checkpoints to find novel therapeutic approaches.

Galectin-9 is one of the galectin family members (6) which is expressed in several tumors (7, 8). Two ICRs bind Galectin-9, including V-domain Ig containing suppressor of T-cell activation (VISTA) and T-cell immunoglobulin and mucin domain 3 (TIM-3) (9, 10). Galectin-9, TIM-3, and VISTA are expressed in both tumor cells and T-cells, where they can act as both receptors and ligands (10-13). The role of VISTA and TIM-3 in T-cell exhaustion in chronic infections and cancers have been investigated in previous studies, and showed that blockade of VISTA led to restoring T-cells in colon cancer models, and over-expression of VISTA on T-cells was observed in multiple myeloma which was associated with tumor progression (13-15). Likewise, it has been observed that the Galectin-9/TIM-3 pathway promoted T-cell exhaustion in chronic hepatitis B infection, and blocking this pathway led to the restoration of HBV-specific T-cells (16). In acute myeloid leukemia (AML), it has been demonstrated that the Galectin-9/VISTA pathway led to pro-apoptotic effects on T-cells via activating granzyme B, and interestingly interaction between Galectin-9 and VISTA did not interfere with binding of TIM-3 to Galectin-9 (11).

Little is known about the role of Galectin-9, TIM-3, and VISTA in B-ALL. In this study, we determined the expression level of Galectin-9, TIM-3, and VISTA in patients with B-ALL and their association with prognosis. The results of this study could be helpful to find new targets for ICB therapy.

## Materials and Methods

***Patients and controls***

Based on previous studies (17-19), we collected 26 B-ALL patients who attended the Hematology and Oncology Clinic of Imam Khomeini Hospital and Bou-Ali Sina Hospital, which is affiliated with Mazandaran University of Medical Sciences, Iran, and 25 healthy controls were included in this study. Patients were diagnosed according to the World Health Organization (WHO) criteria, using blood cell count, morphology, and immunophenotyping of blood cells in peripheral blood or bone marrow samples (20). 

Subjects with chronic viral diseases, including HIV, HBV, and HCV, any congenital or acquired immunodeficiencies, and those with a history of other cancers or any autoimmune diseases were excluded from the study. All study subjects signed the consent form, which was based on the rules of the ethics committee of Mazandaran University of Medical Sciences. A volume of 8–10 ml of heparinized peripheral blood sample was taken from each study subject.

***Isolation of CD8+ T lymphocytes by magnetic-activated cell sorting (MACS)***

**Figure 1:**
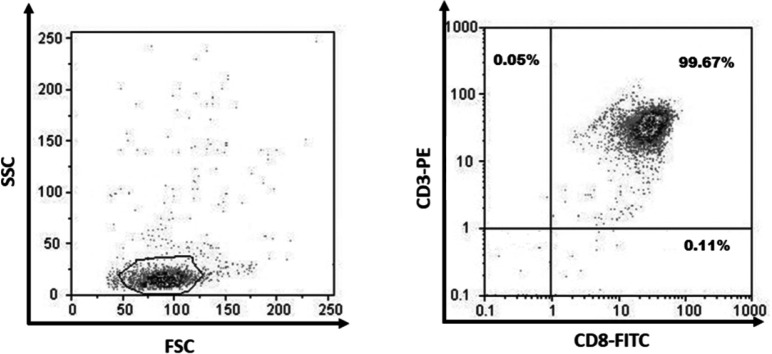
Purity of MACS separated CD8+ T-cells, using anti CD3-PE and anti CD8-FITC antibodies

Peripheral blood mononuclear cells (PBMCs) were isolated from all samples using density gradient centrifugation on Ficoll–Histopaque (Biosera, Nuaille, France). The viability of isolated cells was determined by trypan blue staining. CD8+ T-cells were then purified from PBMCs using magnetic-activated cell sorting (MACS) through CD8 microbeads (Miltenyi Biotec, Germany). To check the purity of isolated CD8+ T-cells, 2 × 10^5^ cells were stained with anti-CD8-FITC (Clone SK1, 0.125 µg/test, eBioscience, San Diego, USA) and anti-CD3-PE (Clone UCHT1, 0.06 µg/test, eBioscience). Appropriate amounts of isotype-matched control antibodies were also used to subtract the background staining. Samples were then analyzed on a Partec PAS flow cytometer system (Partec GmBH, Munster, Germany) using the FlowMax software. As shown in Figure 1, the purity of isolated CD8+ T cells was always more than 99%.

***Flow cytometric analysis***

Isolated CD8+ T-cells (2 × 10^5^ cells) were stained with anti-CD3-PE (Clone UCHT1, 0.06 µg/test, eBioscience) and anti-PD-1-PerCP/Cy5.5 (Clone EH12.2H7, 1 µg/test, Biolegend). Also, to subtract the background staining, appropriate amounts of isotype-matched control antibodies were used. Samples were then analyzed using flow cytometry, as mentioned above.

***Quantitative reverse transcription polymerase chain reaction***

Total RNA was isolated from CD8+ T-cells using the FavorPrep Blood/Cultured Cell Total RNA Mini Kit (Favorgen*,* Taiwan), based on the manufacturer’s protocol. The quantity and quality of isolated RNA were confirmed by nano-spectrophotometer and electrophoresis. Complementary DNA (cDNA) was synthesized using the Yekta-tajhiz cDNA synthesis kit (Yekta tajhiz, Tehran, Iran). Quantitative Reverse Transcription Polymerase Chain Reaction (qRT-PCR) was performed using Real Q Plus 2x Master Mix (High Rox, Ampliqon, Denmark) reagent on an ABI Step one plus Real-Time system (Thermo Scientific) with specific primers for Galectin-9, TIM-3, and, VISTA as well as ACTB, as a housekeeping gene. PCR was performed using 10 pmol of each forward and reverse primer (Table 1), 1 µl of cDNA, 7.5 µl of the Master mix, and 4.5 µl of PCR grade water, and amplified at 95 °C for initial denaturation followed by 45 cycles at 95 °C for 15 sec, 60 °C for 30 sec, and extension at 72 °C for 30 sec. After normalized to ACTB, relative expression levels of Galectin-9, TIM-3, and VISTA, were determined using the 2^-ΔΔCt^ method (21).

***TARGET data analysis***

The mRNA expression profile and clinical information of 154 B-ALL patients were obtained from the Therapeutically Applicable Research To Generate Effective Treatments (TARGET)(https://ocg.cancer.gov/programs/target) database. Detailed patient information was listed.

***Statistical analysis***

Statistical analyses were performed using GraphPad Prism 6 (San Diego, CA, USA) and SPSS16 (North Castle, NY, USA) software packages. Data are presented as Mean±SEM. Mann-Whitney U test and Student’s *t*-test were applied to calculate the mean difference between the groups. Pearson’s rank correlation analysis was used to calculate the correlations between Programmed Cell Death Ligand 1 (PD-L1), Galectin-9, and TIM-3, also for univariate survival analysis, Kaplan–Meier plots with log-rank test were presented using data of B-ALL patients from the TARGET. *P*<0.05 was considered statistically significant.

## Results

***Study population***

A total of 26 B-ALL patients (13 males and 13 females, mean age: 15.08 years) and 25 control subjects (15 males and 10 females, mean age: 21.16 years) were enrolled in the study. Major clinical and laboratory characteristics of patients and controls are summarized in Table 2.

***PD-1 expression in patients and controls***

**Figure 2.**
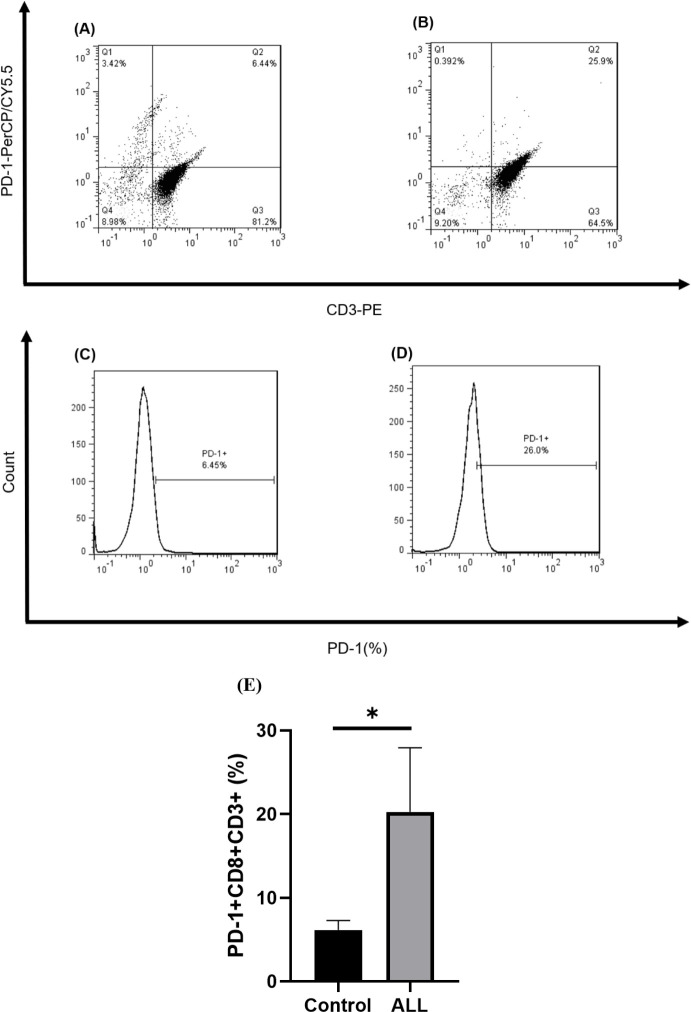
Frequency of exhausted CD8+ T-cells in B-ALL patients and controls

Flow cytometry was applied to enumerate the frequency of PD-1+CD3+ cells among CD8+ T-cells to prove the exhausted phenotype in CD8+ T-cells in B-ALL (Figure 2). PD-1 expression in isolated CD8+ T-cells from patients was significantly higher compared to those from control subjects (*P*=0.011, Figure 2 E).

***Galectin-9, TIM-3, and VISTA mRNA levels in B-ALL patients and control subjects***

**Figure 3:**
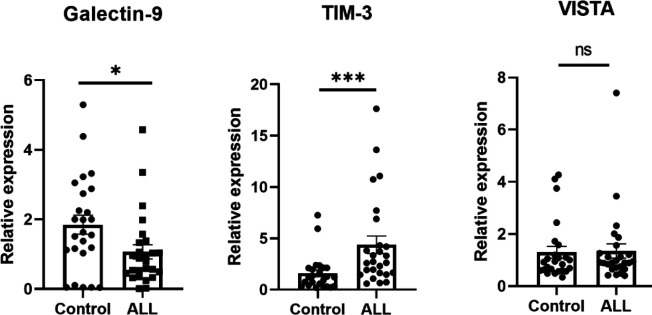
CD8+ T-cells were isolated from the peripheral blood of all study subjects using MACS. mRNA expression of VISTA, Galectin-9, and TIM-3 were then measured using qRT-PCR

Analysis of qRT-PCR data revealed that mRNA expression of Galectin-9 in CD8+ T-cells in B-ALL patients was significantly lower than those in the control group (*P*=0.016). On the other hand, the mRNA level of the TIM-3 gene was significantly higher in CD8+ cells of B-ALL patients, compared to the control group (*P*<0.001). Analysis showed that the VISTA expression was not significantly different between the two study groups (*P*=0.727) (Figure 3).

***Overall survival of patients with high and low expression of Galectin-9 and TIM-3***

**Figure 4:**
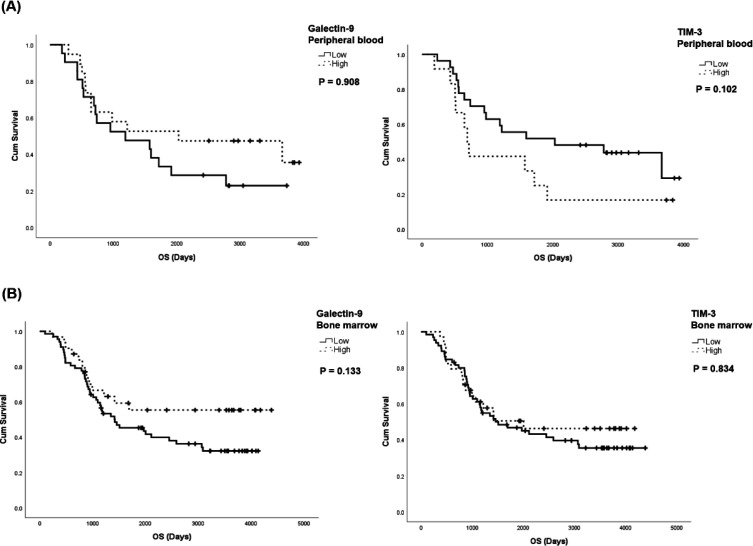
Association between Galectin-9 and TIM-3 expression with OS of B-ALL patients of TARGET database

In order to study the influence of these genes’ expression in leukemic cells on the prognosis of B-ALL patients, bone marrow (BM) and peripheral blood (PB) samples in the TARGET database were divided into two groups of high and low Galectin-9 and TIM-3 expression, according to mean expression levels in each group. Analyses in BM samples showed no significant differences in overall survival (OS) between the two study groups (*P*=0.133 and *P*=0.834, respectively). Moreover, in PB samples, there were no significant differences in the OS of patients with high expression of Galectin-9 and TIM-3 patients with low expression levels (*P*=0.908 and *P*=0.102, respectively) (Figure 4).

***Relapse incidences of patients with high and low expression of Galectin-9 and TIM-3***

**Figure 5:**
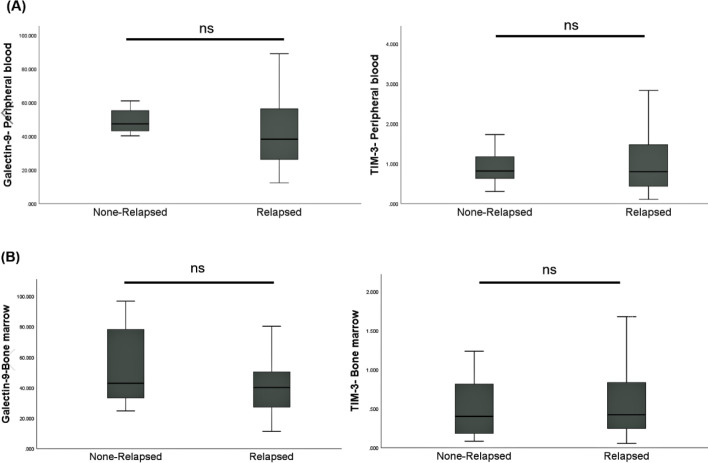
Association of Galectin-9 and TIM-3 expression with patients’ relapse rate

Subsequently, we divided patients into two groups based on incidences of relapse, then compared the expression of TIM-3 and Galectin-9 in leukemic cells in these groups. In bone marrow samples, Galectin-9 and TIM-3 expressions were not significantly different (*P*=0.360 and *P*=0.655, respectively). Besides, in peripheral blood samples, the results showed no significant differences in the expression of Galectin-9 and TIM-3 between those who experienced relapse and patients who did not experience relapse (*P*=0.880 and *P*=0.497, respectively) (Figure 5).

***Correlations in expression of Galectin-9, TIM-3 and PD-L1***

**Figure 6:**
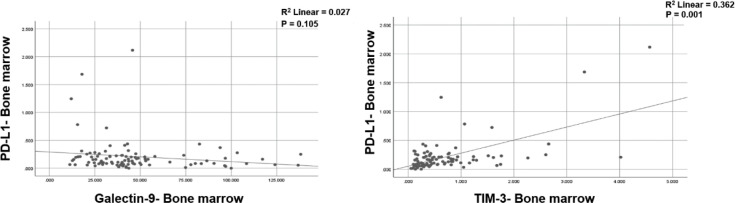
Correlation between Galectin-9, TIM-3, and PD-L1 expression in leukemic cells of BM samples of untreated B-ALL patients

TIM-3: T cell immunoglobulin and mucin domain-containing protein 3; PD-L1: Programmed death-ligand 1; BM: Bone marrow; B-ALL: B-cell Acute lymphoblastic leukemia

Pearson’s correlation analysis was done to explore the potential association between Galectin-9 and PD-L1 expression and we showed that there was no correlation between Galectin-9 and PD-L1 (r = 0.027, *P*=0.105). Moreover, the same analysis was done between TIM-3 and PD-L1 expression which showed a positive correlation (r = 0.362, *P*<0.001). The correlations between Galectin-9, TIM-3, and PD-L1 expression in B-ALL samples were shown in (Figure 6).

**Table 1:** Sequences of forward and reverse primers for the detection of VISTA, Galectin-9, TIM-3, and ACTB genes in patients and control subjects

Gene	Primers (5’-3’)	Product size (bps)
VISTA	F: TCATCCTGCTCCTGGTCTA R: CAGGTGGTGAGGCTTCAA	123
Galectin-9	F: CTGGACAGATGTTCTCTACTC R: AGGAGGATGGACTTGGAT	118
TIM-3	F: CTTTCCAAGGATGCTTACCAC R: TGCTCCGATGTAGATGCCTATT	181
ACTB	F: CCTTCCTGGGCATGGAGTCCT R: TGGGTGCCAGGGCAGTGAT	174

Here is a dot plot chart of a sample which shows a purity of more than 99%.

MACS: Magnetic-activated cell sorting

CD8+ T-cells were obtained from all samples and stained for PD-1 and CD3. PD-1+CD3+ cells in the lymphoid region were defined as exhausted CD8+ T-cells. (A and C) Percent of PD-1+CD8+CD3+ cells in control subjects. (B and D) Percent of PD-1+CD8+CD3+ cells in untreated B-ALL patients. (E) Comparison of PD-1 expression among control subjects (n=10) and untreated B-ALL patients (n=10). Vertical bars represent mean ± SD. *P< 0.05; ** *P*< 0.01; *** *P*<0.001

B-ALL: B-cell Acute lymphoblastic leukemia; PD-1: Programmed death-1

**Table 2:** Major clinical and laboratory characteristics of B-ALL patients

No.	Age (y)	Sex	WBC×10^3^/ mm3	Lymph (%)	PLT×10^3^/ mm3	Hb (g/dL)	Leukemia subgroup
1	19	F	15.5	92.3	55	7	Pre B-ALL
2	1	F	131.6	82.1	19	4.7	Pre B-ALL
3	49	M	12.0	21.0	70	12.4	Pre B-ALL
4	48	M	202.3	38.7	32	7.4	Pre B-ALL
5	69	F	8.9	90.8	76	7	Pre B-ALL
6	51	F	31.0	87.1	19	9.5	Pro B-ALL
7	4	F	5.7	58.1	120	7.9	Pre B-ALL
8	0.5	F	212.62	39.0	70	4.92	Pro B-ALL
9	3	M	129.0	82.8	29	5.5	Pre B-ALL
10	2.5	M	80.0	62.0	87	7.9	Pre B-ALL
11	3	F	7.2	51.8	320	10.9	Pre B-ALL
12	5	M	49.7	32.0	208	3.2	Pre B-ALL
13	8	M	8.4	72.3	157	9.6	Pre B-ALL
14	14	M	14	90.2	37	5.5	pre B-ALL
15	2	F	9.43	84.5	45	9.1	pre B-ALL
16	5	F	29.1	85.3	29	7.3	pre B-ALL
17	12	M	16.6	31	7	7.4	pre B-ALL
18	16	F	21.7	86	49	8	pre B-ALL
19	7	M	5.5	76	48	10.1	pre B-ALL
20	12	F	20.9	38.3	114	13.1	pre B-ALL
21	2	M	5.08	31.9	293	9.9	pre B-ALL
22	6	M	12.98	45.8	210	9.3	pre B-ALL
23	8	F	19.6	93.1	110	7	Pro B-ALL
24	1	M	6.10	45.4	273	10.1	pre B-ALL
25	0.25	F	5.29	14.7	334	9.1	Pro B-ALL
26	2	M	26.97	56	46	11.3	Pre B-ALL

ALL: Acute lymphoblastic leukemia; M: Male; F: Female; WBC: White blood cell count; Lym: Lymphocyte percent in the peripheral blood; PLT: Platelet count; Hb: Hemoglobin

mRNA expressions were first normalized to that of ACTB and then expressed as a fold change as the average expression level of the B-ALL samples divided by that of the control samples. Vertical bars represent mean ± SEM. *P<0.05; ** *P*<0.01; *** *P*<0.001.

MACS: Magnetic-activated cell sorting; VISTA: V-domain Ig suppressor of T cell activation; TIM-3: T cell immunoglobulin and mucin domain-containing protein 3; qRT-PCR: Quantitative reverse transcription polymerase chain reaction; ACTB: Actin-beta; B-ALL: B-cell Acute lymphoblastic leukemia

Kaplan-Meier survival curves of high and low Galectin-9 and TIM-3 expression in PB (A) and BM samples (B) in B-ALL patients. *P*-value was determined by log-rank test.

TIM-3: T cell immunoglobulin and mucin domain-containing protein 3; OS: Overall survival; B-ALL: B-cell Acute lymphoblastic leukemia; PB: Peripheral blood; BM: Bone marrow.

(A) Association of Galectin-9 and TIM-3 expression in leukemic cells of PB samples of relapsed (n = 75) and non-relapsed (n = 24) patients. (B) Association of Galectin-9 and TIM-3 expression in leukemic cells of BM samples of relapsed (n = 32) and non-relapsed (n = 7) patients.

TIM-3: T cell immunoglobulin and mucin domain-containing protein 3; PB: Peripheral blood; BM: Bone marrow

## Discussion

ICB revolutionized the treatment of cancers. Blockades of PD-1 and CTLA-4, in particular, have shown significant effects in the treatment of several solid tumors. However, some patients failed to respond to those therapeutic agents (4). Therefore, the next wave of ICRs, like Galectin-9, TIM-3, and VISTA, has been taken into investigation. A remarkable point about Galectin-9, TIM-3, and VISTA is that they are expressed on both T-cells and tumor cells. Likewise, both TIM-3 and VISTA interact with Galectin-9, where they fantastically play diverse roles as receptors and ligands on CD8+ T-cells (7, 9-11). Therefore, it can be assumed that Galectin-9 TIM-3 and VISTA could induce T-cell exhaustion via autocrine Galectin-9 TIM-3 and VISTA interactions. In this study, we explored the mRNA expression of Galectin-9, TIM-3, and VISTA in CD8+ T-cells in B-ALL patients. 

We first demonstrated that PD-1 expression was significantly higher in CD8+ T-cells from patients than in those from control subjects, indicating that the dominant population of CD8+ T-cells was exhausted. Galectin-9 expression in CD8+ T-cells from B-ALL patients was significantly lower than in those from the control group. Although TIM-3 expression was significantly higher in CD8+ T-cells in B-ALL patients than in those from the control group, VISTA showed no significant difference. Regarding the role of Galectin-9 in T-cell exhaustion, it has been revealed that Galectin-9+ T-cells expressed higher levels of exhaustion-related markers, including Eomesodermin (EOMES), B-lymphocyte-induced maturation protein-1 (Blimp-1), and Glucose transporter1 (Glut1) (10). Moreover, higher expression of TIM-3 and VISTA in T-cells has been observed in several tumors (22) (14). However, based on our results, it seems that TIM-3, but not Galectin-9 and VISTA, could play a critical role in CD8+T-cells exhaustion in B-ALL and might be a remarkable target for ICB and in restoring exhausted T-cells in B-ALL. Concurrently, it has been reported that TIM-3 blockade enhanced IFN-γ expression by CD8+ T-cells in a murine orthotopic mammary carcinoma model and reduced the population of MDSCs in head and neck squamous cell carcinoma (23, 24).

Galectin-9 expression was determined in leukemic cells in AML and chronic lymphoblastic leukemia (11, 25), also, TIM-3 overexpression in BM and PBMCs in ALL patients including B- and T-ALL has been previously reported (26); while the prognostic role of Galectin-9 and TIM-3 in B-ALL has not been investigated. We thus assessed the association between the expression of TIM-3 and Galectin-9 in leukemic cells based on the data in the TARGET database. We did not include VISTA in our bioinformatics assessment since there is no evidence that B-cells express VISTA (27). Our results showed no significant association between the expression of Galectin-9 and TIM-3 in leukemic cells in both PB and BM samples and the patients’ OS. Moreover, we evaluated whether relapse incidences were associated with TIM-3 and Galectin-9 expression in leukemic cells. The results demonstrated that TIM-3 and Galectin-9 expressions in PB and BM samples were not significantly different between relapsed and non-relapsed patients.

It has been revealed that Galectin-9 expression was correlated with disease progression and lower OS in gliomas. However, in some other tumors, like hepatocellular carcinoma, there was no association between Galectin-9 and survival rate (28, 29). Moreover, increased TIM-3 expression in leukemic cells in AML was shown to be associated with poor prognosis and lower survival rates (30). In line with the latter study, TIM-3 was shown to be over-expressed in colon cancer which was correlated with lymphatic metastasis and lower survival rate (31). However, based on our results, high expression of Galectin-9 and TIM-3 in leukemic cells did not associate with prognosis of B-ALL. 

We next utilized PD-L1 expression on leukemic cells as a predictive biomarker which has also been investigated in previous studies (32). We assessed the correlation between TIM-3 and Galectin-9 with PD-L1, which revealed a positive correlation between TIM-3 and PD-L1 expression.

Our study had some limitations, like we did not validate data from TARGET and, we did not evaluate protein expression of Galectin-9, TIM-3, and VISTA.

## Conclusion

 In summary, our results indicate a role for TIM-3, as an ICR, in B-ALL. Given that Galectin-9, TIM-3, and VISTA could simultaneously express on a T-cell. Besides the possible synergistic effect of Galectin-9/VISTA and Galectin-9/TIM-3 pathways, it can be assumed that Galectin-9, TIM-3, and VISTA might contribute to T-cell exhaustion through an autocrine pathway. Based on this assumption and according to significant increase of TIM-3 in CD8+ T-cells, it seems that remarkable over-expression of TIM-3 could compensate for lower Galectin-9 expression within the Galectin-9/TIM-3 pathway and this pathway could be a candidate pathway for ICB in B-ALL.

## Statement of Ethics

All study subjects have given their written informed consent. The study protocol was approved by the Ethical Committee of Mazandaran University of Medical Sciences.

## Authors’ Contributions

All authors contributed to the study. A A, M T, H AO, and R V designed and conducted the research. A A carried out the assays. A A, MM D, and A P contributed to data collection and analysis. H K, M N, and S S provided the samples. A A, M T, A K, and A N prepared the manuscript. All authors read and approved the final manuscript.

## Conflicts of Interest

The authors have no conflicts of interest to declare.
